# Advancements in Sensors and Analyses for Emotion Sensing

**DOI:** 10.3390/s24134166

**Published:** 2024-06-27

**Authors:** Wataru Sato

**Affiliations:** Psychological Process Team, Guardian Robot Project, RIKEN, 2-2-2 Hikaridai, Seika-cho, Soraku-gun, Kyoto 619-0288, Japan; wataru.sato.ya@riken.jp

## 1. Introduction

Exploring the objective signals associated with subjective emotional states has practical significance. Emotional experiences play a fundamental role in people’s well-being [1] and in business success, such as in the development of new products [2]. However, subjective ratings of emotion have several limitations. First, the scientific rigor of self-reports is controversial [3]. Second, subjective ratings can be biased with respect to demand characteristics [4] and social desirability [5]. Third, dynamic, continuous ratings are difficult while performing active tasks [6]. Fourth, specific individuals, such as those with alexithymia [7], those with dementia [8], and young children [9], have difficulty evaluating emotional experiences. Finally, bodily and behavioral emotional responses may be elicited without subjective experience [10]. Measurements of objective signals associated with emotional experiences complement subjective ratings by providing objective, unbiased, dynamic, monitorable, and subtle emotional information.

Many studies have developed emotion-sensing methods based on physical or physiological signals [11]. For example, previous studies reported that facial expressions [12] and autonomic nervous system activity [13] reflect the subjective experiences of basic emotional categories. Other studies reported that facial electromyography (EMG) and electrodermal activity (EDA) reflect the dimensions of emotional valence and arousal, respectively [14]. Several wearable devices have been developed to implement emotion sensing in real-life situations [15], and various machine-learning (ML) models have been proposed to analyze these emotion-related data [16].

However, several limitations remain in current emotion-sensing methods. For example, whether emotion categories could be estimated using facial expressions [17] or physiological signals [18] is controversial. The magnitude of the correlations between facial EMG activity and subjective valence experiences is reportedly weak in some studies (e.g., ref. [19]). Only a small proportion of wearable emotion-sensing devices have been empirically validated [20]. Several challenges for emotion analysis have been pointed out, including an advanced analysis of multimodal and real-life data [16].

The present Special Issue brings together a collection of new articles that have explored new sensors or analyses for emotion sensing aimed at overcoming these limitations. These articles are introduced in the following sections.

## 2. Advancements in Sensors

Some of the studies in this Special Issue explored new sensors or measures for emotion sensing. Hsu and Sato [21] validated a video-based automated facial action analysis by simultaneously recording the video data of facial expressions and facial EMG. The results indicated positive correlations between automated facial action signals and facial EMG, although the sensitivity of the former signals was weaker than the latter. To identify new measures for emotion sensing, Sato and Kochiyama [22] recorded EMG from the facial and trapezius muscles, fingertip temperature, and dynamic ratings of subjective emotional valence and arousal while participants viewed emotional film clips. Valence ratings were linearly associated with corrugator and zygomatic EMG; fingertip temperature changes were associated with dynamic arousal ratings, while trapezius EMG was unrelated to either valence or arousal ratings. These results suggest that new measures, including automatically analyzed facial action units and fingertip temperature, are useful for emotion sensing.

Several studies in this Special Issue tested the effectiveness of wearable devices for implementing emotion sensing in real-life situations. Balconi et al. [23] recorded several autonomic nervous system activity measures, including pulse volume amplitude, using a portable system—alongside behavioral and self-reported data—in visually impaired and sighted participants during product selection in a grocery store. Pulse volume amplitudes correlated negatively with time spent on product identification and positively with simplicity in finding products among the entire sample, suggesting that the physiological signals reflected positive emotional reactions. Algumaei et al. [24] recorded heart rate (HR) signals using wearable sensors and measured task performance and subjective frustration levels during a collaborative computer-based simulation task among three members. Some measures of physiological synchrony estimated based on HR signals revealed associations with task performance and subjective frustration level. Arabian et al. [25] recorded EDA and HR signals from wearable sensors on the hand and chest, respectively, while participants observed emotional images. ML modeling for features selected from these signals effectively identified the emotional states. Wang et al. [26] detected driver fatigue based on dual-channel electroencephalography (EEG) and HR signals recorded using a portable device. The analysis of the datasets demonstrated that an EEG-based real-time evaluation of driving fatigue status is possible. Onishi et al. [27] measured children’s behavior in story-telling activities using wearable motion sensors attached to their heads. The children’s curiosity level could be estimated based on their head motion data. These findings reveal that recording physiological or physical signals using wearable devices provides useful information about emotional states in real-life situations.

In summary, these studies suggest that further exploration of emotion-sensing sensors is a promising line of research.

## 3. Advancements in Analyses

Several studies in this Special Issue reported new analyses aimed at improving emotion sensing. John and Kawanishi [28] proposed a novel method for analyzing multimodal information to address missing modality and multimodal portability problems. Experiments using existing datasets validated the proposed method, showing better accuracy of emotion classification from audio-visual data than other methods. Wang et al. [29] proposed a new feature extraction method using the amplitude level quantization technique to analyze HR variability for emotion sensing. The emotion classification accuracy of this method surpassed existing techniques. Bahameish et al. [30] proposed robust strategies to reduce the effects of overfitting and data leakage in ML modeling using HR variability for stress detection. Their analysis revealed a model that accurately differentiated stress from neutral states with high generalizability. Xu et al. [31] proposed a network model based on a gated recurrent unit and multi-head attention for speech emotion recognition. The experimental results showed that this model had better classification performance for speech emotion than commonly used models. Li et al. [32] proposed a method to add perturbation to generate diverse emotional responses in dialogue systems, which can be useful for emotion recognition. The experimental results showed that the method improved the accuracy and diversity of emotional expressions compared with baseline methods. These studies illustrate the development of new analyses to enhance the efficacy and usefulness of emotion sensing using physical and physiological signals.

## 4. Conclusions

The findings in this Special Issue illustrate recent advancements in sensors and analyses for emotion sensing. Subjective emotional states can be more accurately estimated in more realistic situations with improved sensors and analyses. Understanding the associations between subjective emotional and physical/physiological states is also theoretically important and has been of great interest because it can teach us the psychological mechanisms underlying subjective emotional experiences [33]. Further research is warranted to investigate emotion-sensing methods using objective signals.
